# Imbalances in authorship, geographic and institutional contexts, and funding sources in research on gender approaches to sexual and reproductive health in Africa: a scoping review

**DOI:** 10.1080/26410397.2026.2616137

**Published:** 2026-01-16

**Authors:** Woldekidan Amde, Kéfilath Bello, Tanya Jacobs, Tk Sundari Ravindran, Asha S. George

**Affiliations:** a Researcher, School of Public Health, University of the Western Cape, Cape Town, South Africa.; b Researcher, Centre de Recherche en Reproduction Humaine et en Démographie, Cotonou, Benin; c Independent Researcher, Independent Researcher, Cape Town, South Africa; d Independent Researcher, Independent Researcher, Trivandrum, India; e South African Research Chair in Health Systems, Complexity and Social Change, School of Public Health, University of the Western Cape, Cape Town, South Africa

**Keywords:** epistemic injustice, gender equality, gender transformative approach, global health, research leadership, sexual and reproductive health, sexual and reproductive health and rights

## Abstract

National research leadership is critical for generating locally responsive knowledge, especially grounded in gender approaches, given its engagement with local social contexts. We conducted a focused analysis of a scoping review to examine patterns in authorship, geographic and institutional contexts, and funding sources, in studies that apply gender approaches to sexual and reproductive health (SRH) across Africa. The review examined 45 publications in PubMed and Scopus (2012–2022) and included consultation with African gender and health experts. Our analysis revealed unequal distribution of papers across sub-regions in Africa (48.9% were based in Southern Africa, 37.8% in Eastern Africa, 11.1% in Western Africa, and 2.1% in Northern Africa). The distribution of articles by first and last authors’ country of residence depicted disparity between authors in high-income countries and those in Africa, and between authors based in South Africa and those stationed in the rest of Africa (USA 46.7%, Europe 17.8%, Canada 2.2%, South Africa 22.2%, and the rest of Africa 11.1%). Similarly, unequal patterns exist regarding the distribution of last authors (USA 42.9%, Europe 9.5%, Canada 4.8%, South Africa 28.6%, and the rest of Africa 14.3%). One-fifth of the papers feature no local authors. Funding sources show a stark difference, with just 9.4% of the funding coming from Africa, exclusively South Africa, and the rest originating from high-income countries (USA 36.5%, UK 14.1%, Canada 8.2%, and Sweden 5.9%). The authors call for ensuring local ownership and leadership of research in Africa, increasing domestic investment and addressing disparities across sub-regions.

## Plain language summary

National research leadership is crucial for generating knowledge that is locally responsive. This is particularly important in research that seeks to advance gender equality, specifically, in sexual and reproductive health and rights. We examined four aspects of primary research in the field across Africa. These include geographic setting, authorship representation, institutional background and funding sources. Our scoping review found 45 primary research articles from prominent public health and social science databases from 2012 to 2022 that revealed unequal patterns in authorship, geographic and institutional contexts, and funding sources. The reviewed papers were situated in Southern Africa (48.9%), Eastern Africa (38%), Western Africa (11.1%), and Northern Africa (2.1%). Disparities prevail in the distribution of authors by country. Of the total, 67% of the first authors and 57% of the last authors were not based in Africa. One-fifth of the papers feature no local authors. At the same time, South African authors make up 22% of the first authors and 29% of the last authors. These figures for South Africa are double the proportion for the authors from the rest of Africa. Under 10% of the research funding came from Africa, exclusively from South Africa. The remaining originates from high-income countries. The authors consulted with African gender and health experts to contextualise the findings. The consultation highlighted the local and global factors contributing to these imbalances. The authors call for ensuring local ownership and leadership of research in Africa. Increasing domestic investment and addressing disparities across sub-regions are also critical.

## Résumé

Le leadership national en matière de recherche est essentiel pour produire des connaissances adaptées au niveau local, spécialement dans le domaine de la recherche sur le genre, compte tenu de ses liens avec les contextes sociaux locaux. Nous avons mené une analyse ciblée d'une étude de la portée afin d'examiner les tendances en matière d'auteurs, de contextes géographiques et institutionnels, et de sources de financement, dans les études qui appliquent une perspective de genre à la santé sexuelle et reproductive (SSR) en Afrique. Cette analyse a porté sur 45 publications dans PubMed et Scopus (2012 – 2022) et a inclus des consultations avec des experts africains du genre et de la santé. Notre analyse a révélé une répartition inégale des articles entre les sous-régions africaines (48,9% provenaient d'Afrique australe, 37,8% d'Afrique de l'Est, 11,1% d'Afrique de l'Ouest et 2,1% en Afrique du Nord). La répartition des articles selon le pays de résidence du premier et du dernier auteur montre une disparité entre auteurs des pays à revenu élevé et ceux d'Afrique, ainsi qu'entre les auteurs basés en Afrique du Sud et ceux du reste du continent (États-Unis 46,7%, Europe 17,8%, Canada 2,2%, Afrique du Sud 22,2% et reste de l'Afrique 11,1%). De même, la répartition des derniers auteurs dénote des inégalités (États-Unis 42,9%, Europe 9,5%, Canada 4,8%, Afrique du Sud 28,6% et reste de l'Afrique 14,3%). Un cinquième des articles ne comptaient aucun auteur local. Les sources de financement montrent une différence marquée, avec à peine 9,4% des fonds provenant d'Afrique, exclusivement d'Afrique du Sud, le reste ayant son origine dans des pays à revenu élevé (États-Unis 36,5%, Royaume-Uni 14,1%, Canada 8,2% et Suède 5,9%). Les auteurs appellent à garantir l'appropriation et le leadership locaux en matière de recherche en Afrique, à accroître les investissements nationaux et à corriger les disparités entre sous-régions.

## Resumen

El liderazgo nacional en investigación es fundamental para generar conocimiento receptivo a nivel local, especialmente en investigación de género, dada su compromiso con contextos sociales locales. Realizamos un subanálisis de una revisión de alcance para examinar los patrones en contextos de autoría, geográficos e institucionales, así como las fuentes de financiación de estudios que aplican una perspectiva de género a la salud sexual y reproductiva (SSR) en África. examinarla revisión examinó 45 publicaciones en PubMed y Scopus (2012 – 2022) y consultar con expertos africanos en género y salud. Nuestro análisis reveló una distribución desigual de artículos en diferentes subregiones de África (48.9 % estaban basados en África Meridional, 37.8 % en África Oriental, 11.1 % en África Occidental y 2.1% en África Septentrional). La distribución de artículos por país de residencia del primer y último autor mostró disparidad entre autores con sede en países de altos ingresos y aquellos en África, y dentro del continente entre autores con sede en África Meridional y aquéllos que residían en otras regiones de África (EE. UU. 46.7 %, Europa 17.8%, Canadá 2.2 %, África Meridional 22.2 % y el resto de África 11.1 %). Además, existen patrones desiguales con relación a la distribución de últimos autores (EE. UU. 42.9 %, Europa 9.5 %, Canadá 4.8 %, Sudáfrica 28.6 % y el resto de África 14.3 %). Una quinta parte de los artículos no mencionan autores locales. En lo que respecta a las fuentes de financiación, existe una marcada diferencia; solo el 9.4 % de la financiación proviene de África, exclusivamente de Sudáfrica, y el resto proviene de países de altos ingresos (EE. UU. 36.5 %, Reino Unido 14.1 %, Canadá 8.2 % y Suecia 5.9 %). Los autores piden que se garantice la propiedad intelectual y el liderazgo de las investigaciones a nivel local en África, y que se aumenten las inversiones nacionales y se aborden las disparidades entre subregiones.

## Introduction

Progress towards sexual and reproductive health and rights (SRHR) is integral to SDG 3 (good health and wellbeing) and SDG 5 (gender equality and women’s empowerment).^1^ However, the pace of transformation in Africa, particularly in West Africa, is far slower than expected.^2–4^ Maternal mortality has stagnated and even reversed in some African contexts.^5^ This is despite known effective interventions, including education on sexual and reproductive health (SRH), contraception, condom use, HIV/STI prevention, and gender-based violence.^6^

Underpinning this lack of progress are stark and persistent gendered inequalities. Take, for example, the proportion of women aged 15–49 years who make their own informed decisions regarding sexual relations, contraceptive use and reproductive health care. The figures for South Africa and Ghana are 64.9% and 52.0%, respectively, in contrast to 7.3% and 20.3% for Niger and Burkina Faso.^7^

This dire situation in the continent is bound to be further pronounced as gains made thus far are decimated by the aftermath of the COVID pandemic,^8^ and the spiralling effects of climate change^9,10^ combined with drastic and abrupt funding cuts.^11^ The continent is expected to lose 70% of its Official Development Assistance^12^ in what is described as “geopolitical vandalism”.^13^ Worsening debt crises are also resulting in increasing austerity measures that undermine public sectors and services, including health and education.^12,14^ In addition, the backlash against gender equality and SRHR^14^ is stalling or reversing legislative protections for key services. Particularly problematic are nativist calls that claim that sexual and reproductive health and rights are not African, and that the universal principles of gender equality and human rights reflect Western artifices and colonial legacies.^15^

Against this background, the application of a gender approach in research and programming (i.e. integrating a gender lens in research and interventions, including gender-responsive, gender-transformative, and gender-specific approaches) to address the root causes of intersectional gender inequality remains even more salient as a critical way of supporting the realisation of SRHR.^16–18^ At the same time, the role and impact of research and programming that employ gender approaches remain contested. Dworkin et al. highlight its incremental contribution^19^ while critics argue that it is skewed towards generic, short-term, micro-level changes vs sustaining long-term structural change adapted to local contexts.^19–22^ Concurrently, scholarship on gender and health does not escape blame for perpetuating global epistemic discrimination and for not sufficiently engaging and elevating Global South authors and policy makers, who are critical for effective and sustained knowledge generation and translation.^17^ Given how effective global campaigns have been in fomenting backlash against gender equality,^23^ this lack of local ownership of research guided by gender approaches is problematic.

Research has highlighted problematic patterns, often referred to as “authorship parasitism”, “stuck in the middle”, or “authorship tokenism”, which point to the complete absence, inadequate recognition, or superficial inclusion of local researchers.^24,25^ Such patterns reflect broader systemic challenges across the entire research ecosystem. Bhakuni and Abimbola map this crisis into different and often unexamined modalities of “epistemic wrongs/injustice” that systemically isolate knowledge producers and users in the Global South, across the different spaces of the knowledge system, from research partnerships, research framing and authorship, with long-term impacts on the local ownership, local research capacity, and relevance of research.^26–28^ Particularly important, given the lack of implementation of programmes addressing gender equality, is the need for African thought leadership supporting and advocating that these implementation gaps and outstanding inequalities be addressed. Understanding and addressing these dynamics constraining Global South research leadership is a critical stepping stone for more effective health programmes and policies.^29–33^

While other reviews have examined inequalities in scholarship across knowledge platforms, health issues or research partnerships,^28–30,32,34^ to date, there has been no systematic examination of such inequalities in scholarship related to integrated gender approaches to SRH research in Africa. Mattison et al. shed light on patterns in SRHR research, examining its focus and distribution of co-authorship networks across countries, but their analysis has a global focus.^35^ We conducted this focused analysis of a scoping review on SRH research across Africa that integrates gender approaches, examining patterns of leadership in authorship, geographic and institutional contexts, and funding sources. This complements the main scoping review^36^ that primarily sets out to take stock of what is documented in terms of researches that apply a gender lens to SRH across the African continent.

## Methods

The scoping review focused on publications in major public health and social science databases (PubMed and Scopus) between 1 January 2012 and 12 December 2022. The review was informed by Arksey and O’Malley’s guide for scoping reviews, which encompasses stages of developing research questions, locating and identifying pertinent papers, extracting and charting data, analysis, synthesis and reporting, and an optional consultation to improve methodological rigour.^37^

### Search strategy

The authors developed a search strategy that combined five themes: (1) Application of a gender lens, including gender-responsive, gender-transformative, and gender-specific approaches; (2) Sexual and reproductive health, including sexual health, sexual rights, reproductive health, reproductive rights, sexual and reproductive health and rights, maternal health, abortion, family planning, contraception, gender-based violence*, HIV, STI, breast cancer, cervical cancer, and menstrual hygiene;^1^ (3) Focus on African countries; (4) Interventions, including intervention, program*, strategy*, and plan*; (5) Emphasis on measurement, including monitor*, evaluat*, and assess*.

The search strings were refined in consultation with the university librarian and a resource person at the School of Public Health, University of the Western Cape. After the initial search, the fifth set of measurement-related terms was excluded in order to broaden the scope and yield of the search. This modification increased the number of PubMed database search results from 87 to 159. The final search strings used are presented below:

PUBMED:

TITLE/ABSTRACT – Excluding (monitor* OR evaluat* OR assess*) 2012–2022 = 159 Results

(gender-responsive) OR (“gender transformative”) OR (“gender specific”) AND (intervention OR program* OR strateg* OR plan*) AND (“sexual health” OR “sexual rights” OR “reproductive health” OR “reproductive rights” OR “sexual and reproductive health and rights” OR “maternal health” OR abortion OR “family planning” OR contraception OR “gender-based violence*” OR HIV OR STI OR “breast cancer” OR “cervical cancer” OR “menstrual hygiene”) AND (Africa OR Algeria OR Angola OR Benin OR Botswana OR “Burkina Faso” OR Burundi OR “Cape Verde” OR Cameroon OR “Central African Republic” OR Chad OR Comoros OR Congo OR “Democratic Republic of the Congo” OR “Cote d’Ivoire” OR Djibouti OR Egypt OR “Equatorial Guinea” OR Eritrea OR Eswatini OR Ethiopia OR Gabon OR Gambia OR Ghana OR Guinea OR Guinea-Bissau OR Kenya OR Lesotho OR Liberia OR Libya OR Madagascar OR Malawi OR Mali OR Mauritania OR Mauritius OR Morocco OR Mozambique OR Namibia OR Niger OR Nigeria OR Rwanda OR “Sao Tome and Principe” OR Senegal OR Seychelles OR “Sierra Leone” OR Somalia OR “South Africa” OR “South Sudan” OR Sudan OR Tanzania OR Togo OR Tunisia OR Uganda OR Zambia OR Zimbabwe)

### Study selection

The search results were uploaded onto Covidence for screening. Once duplicate references were removed, title and abstract screening for each article was done independently by two members of the reviewing team following inclusion and exclusion criteria (WA, KB, TJ, SR, AG). Publications were deemed relevant if they involved gender approaches in research and programming on SRH in Africa (see Table 1 for a complete list of inclusion and exclusion criteria). Any conflict in the inclusion of references between reviewers was discussed and resolved during regular debrief meetings. The full text of all articles deemed relevant based on the title and abstract screening was retrieved, and each article was reviewed by a reviewing team member (WA, KB, TJ, SR, AG).

**Table 1.** Inclusion criteria

Criteria	Inclusion criteria
Study Type	Peer-reviewed primary research articles published in journals
Publication date	Year: 2012–2022
Gender Focus	Focuses on gender (gender-responsive, gender-transformative or gender-specific)
Intervention focus	Focuses on intervention (intervention, programme, strategy, or planning)
SRHR Focus [1]	Focuses on SRHR issues: sexual health sexual rights reproductive health reproductive rights sexual and reproductive health and rights maternal health abortion family planning contraception gender-based violence HIV STI breast cancer cervical cancer menstrual hygiene
**Geographic focus**	Focuses on Africa, or any of the 54 African countries
**Language**	English and French

### Data extraction

The review team developed and piloted a data extraction Excel spreadsheet to chart a wide range of data. Those related to this paper include details about the first and last authors (name, country of residence, organisational affiliation), total number of authors, total number of local authors, funding sources, and countries where the study was situated (Table 2 and Supplementary Table S1).

**Table 2.** Summary table of identified articles

S/No	Author	Year	Title of the article	First author's country	Last author's country	Country/ ies that the study is situated in
1.	[38]	2014	Transforming gender roles in domestic and caregiving work: preliminary findings from engaging fathers in maternal, newborn, and child health in Rwanda	USA	USA	Rwanda
2.	[39]	2018	“I Tried to Resist and Avoid Bad Friends”: The Role of Social Contexts in Shaping the Transformation of Masculinities in a Gender Transformative and Livelihood Strengthening Intervention in South Africa	South Africa	South Africa	South Africa
3.	[40]	2018	MenCare + in South Africa: findings from a gender transformative young men’s group education on sexual and reproductive health and rights	Netherlands	South Africa	South Africa
4.	[41]	2018	A gender assessment of Malawi’s National Nutrition Policy and Strategic Plan 2007–2012	South Africa	USA	Malawi
5.	[42]	2022	A Family–Focused, Sibling–Synchronous Intervention in Borno State, Nigeria: Exploring the Impact on Family Functioning and Household Gender Roles	USA	USA	Nigeria
6.	[43]	2015	Lessons learned from engaging men in sexual and reproductive health as clients, partners and advocates of change in the Hoima district of Uganda	South Africa	South Africa	Uganda
7.	[44]	2020	Constructing, reproducing and challenging masculinities in a participatory intervention in urban informal settlements in South Africa	South Africa	South Africa	South Africa
8.	[45]	2020	Support or control? Qualitative interviews with Zambian women on male partner involvement in HIV care during and after pregnancy	USA	USA	Zambia
9.	[46]	2022	Opportunities and challenges in preventing violence against adolescent girls through gender transformative, whole – family support programming in Northeast Nigeria	USA	USA	Nigeria
10.	[47]	2016	Gendered power dynamics and women’s negotiation of family planning in a high HIV prevalence setting: a qualitative study of couples in western Kenya	USA	USA	Kenya
11.	[48]	2021	A cluster randomised controlled trial to evaluate the impact of a gender transformative intervention on intimate partner violence against women in newly formed neighbourhood groups in Tanzania	UK	UK	Tanzania
12.	[49]	2021	Impact of a cash plus intervention on gender attitudes among Tanzanian adolescents	Ireland	USA	Tanzania
13.	[50]	2020	Re-conceptualising gender and power relations for sexual and reproductive health: contrasting narrative of tradition,unity, and rights	USA	USA	Malawi
14.	[51]	2019	Measuring sexual relationship power equity among young women and young men south Africa:Implications for gender-transformative programming	Canada	Canada	South Africa
15.	[52]	2020	Effective prevention of intimate partner violence through couples training: a randomised controlled trial of Indashyikirwa in Rwanda	South Africa	USA	Rwanda
16.	[53]	2012	Impact of a Gender-Transformative HIV and Anti-violence Program on Gender Ideologies and Masculinities in Two Rural, South African Communities	USA	South Africa	South Africa
17.	[54]	2020	Leaving No Man Behind: How Differentiated Service Delivery Models Increase Men’s Engagement in HIV Care	South Africa	South Africa	South Africa
18.	[55]	2015	Reconstructing masculinity? A qualitative evaluation of the Stepping Stones and Creating futures interventions in urban informal settlements in South Africa	South Africa	South Africa	South Africa
19.	[56]	2017	Evaluation of a male engagement intervention to transform gender norms and improve family planning and HIV service uptake in Kabale, Uganda	USA	Uganda	Uganda
20.	[57]	2021	Impact of home visits to pregnant women and their spouses on gender norms and dynamics in Bauchi State, Nigeria: Narratives from visited men and women	Nigeria	Canada	Nigeria
21.	[58]	2021	Policy foundations for transformation: a gender analysis of adolescent health policy documents in South Africa	South Africa	South Africa	South Africa
22.	[59]	2021	Gendered health, economic, social and safety impact of COVID-19 on adolescents and young adults in Nairobi, Kenya	USA	Belgium	Kenya
23.	[60]	2018	Community mobilisation to modify harmful gender norms and reduce HIV risk: results from a community cluster randomised trial in South Africa	USA	South Africa	South Africa
24.	[61]	2020	Effectiveness of a culturally appropriate intervention to prevent intimate partner violence and HIV transmission among men, women, and couples in rural Ethiopia: Findings from a cluster-randomised controlled trial	USA	Ethiopia	Ethiopia
25.	[62]	2019	Masculinity and engagement in HIV care among male fisherfolk on HIV treatment in Uganda	USA	USA	Uganda
26.	[63]	2020	Enhancing agency for health providers and pregnant women experiencing intimate partner violence in South Africa	USA	USA	South Africa
27.	[64]	2021	Fathers and grandmothers experiences participating in nutrition peer dialogue groups in Vihiga County, Kenya	Kenya	USA	Kenya
28.	[65]	2020	Fostering gender equality and alternatives to violence: perspectives on a gender-transformative community mobilisation programme in rural South Africa	USA	South Africa	South Africa
29.	[66]	2016	Integrating theory-based evaluation and process tracing in the evaluation of civil society gender budget initiatives	Belgium	Belgium	Uganda
30.	[67]	2021	Tactical Activism: Religion, Emotion, and Political Engagement in Gender Transformative Interventions	Spain	NA	South Africa, Mozambique, DRC
31.	[68]	2016	Refugees and “host communities’ facing genderbased violence: developing an area-based approach to gender-based violence around Mbera Camp, Mauritania	Senegal	NA	Mauritania
32.	[69]	2022	How gender norms and “good girl” notions prevent adolescent girls and young women prevent adolescent girls and young women from engaging with PrEP: qualitative insights from Zimbabwe	Denmark	Zimbabwe and UK	Zimbabwe
33.	[70]	2013	Understanding the meanings of male partner support in the adherence to therapy among HIV-positive women: a gender analysis	Italy	Italy	Malawi
34.	[71]	2015	“One Man Can”: shifts in fatherhood beliefs and parenting practices following a gendertransformative programme in Eastern Cape, South Africa	South Africa	USA	South Africa
35.	[72]	2013	Lessons learned: program messaging in gender-transformative work with men and boys in South Africa	USA	South Africa	South Africa
36.	[73]	2020	Tell them you are planning for the future: Gender norms and family planning among adolescents in northern Uganda	USA	USA	Uganda
37.	[74]	2012	Sexuality and the limits of agency among South African teenage women: Theorising femininities and their connections to HIV risk practises	South Africa	South Africa	South Africa
38.	[75]	2021	Community-level spillover effects of an intervention to prevent intimate partner violence and HIV transmission in rural Ethiopia	USA	USA	Ethiopia
39.	[76]	2021	Measuring Men’s Gender Norm Beliefs Related to Contraception: Development of the Masculine Norms and Family Planning Acceptance Scale	USA	USA	Kenya
40.	[77]	2016	What role can gender-transformative programming for men play in increasing men’s HIV testing and engagement in HIV care and treatment in South Africa?	USA	USA	South Africa
41.	[78]	2021	A Promising Approach to Preventing Gender-Based Violence and HIV Among Slum-Dwelling Youth in Nairobi, Kenya	USA	Kenya	Kenya
42.	[79]	2017	Prevention of mother to child transmission of HIV in Tanzania: assessing gender mainstreaming on paper and in practice	Tanzania	Tanzania	Tanzania
43.	[80]	2021	Adaptation of a gender-transformative sexual and reproductive health intervention for adolescent boys in South Africa and Lesotho using intervention mapping	UK	South Africa	South Africa; Lesotho
44.	[81]	2021	Does Spousal Engagement Improve Cervical Cancer Screening Discussions and Uptake? Lessons from a Before-After Study in a Rural Nigerian Community	Nigeria	Nigeria	Nigeria
45.	[82]	2019	Girl Empower – A gender transformative mentoring and cash transfer intervention to promote adolescent wellbeing: Impact findings from a cluster-randomised controlled trial in Liberia	USA	USA	Liberia
A more comprehensive data charting table on the content of the reviewed paper is included in the main scoping review by George et al. (2025), available at https://www.ajrh.info/index.php/ajrh/article/view/5693/2271						

We used the first institutional affiliation of authors as a proxy for the country of residence of authors. These affiliations were categorised based on the World Bank’s income bracket classification of the countries in which they were situated.^83^ It was not possible to determine the national origin of the authors or the contribution of the African diaspora.

Quality of extracted data and codes/categories was conducted during regular team debriefs, and one of the reviewers (SR) conducted further consultation for data extraction to ensure any discrepancies were addressed and nuanced details were captured.

### Consultation workshop with experts

Given the South African dominance within our research team, consulting with a broader regional and multi-stakeholder group of African experts was deemed key to ensuring our findings – particularly regarding imbalances in authorship, geographic and institutional context, and funding sources – resonated with others on the continent. A consultation workshop is also integral to the scoping review approach and is a purposeful way of generating a broader perspective and enhancing methodological rigour.^37^ We thus conducted the consultation with the specific purpose of validating the findings from the review, uncovering any gaps, and making sense of these by drawing on insights based on the participants’ expertise and grounded experience.

Findings on imbalances in authorship were shared and discussed on 9 May 2024 with experts who were convened for a feminist pan-African workshop that brought together feminists leading the movement for gender equality and SRHR across various spheres – including research, teaching, programming, and activism – against all forms of social, economic, and political discrimination based on sexual or gender identity and other intersecting factors such as disability. The workshop, titled Feminist Fiesta, included 15 purposefully selected feminist researchers, academics, implementers, and activists from 12 countries and 15 institutions in Africa. Careful consideration was given to ensure intersectional, generational, regional, language, and sectoral representation among the participants (see Table 3). Beyond consultation on the broader research project, the Feminist Fiesta workshop sought to build understanding of gender approaches to SRH research, advance decolonisation of global health through African-led scholarship and ownership, and strengthen networks and solidarity among participants and institutions.

**Table 3.** Geographic and discipline profile of workshop participants

Participant ID	Country	Role, disciplinary orientation
P1	Burkina Faso	Researcher, political and social science, demography
P2	Niger	Researcher, medicine, anthropology
P3	Ghana	Researcher and policy analyst, public health
P4	Rwanda	Civil society director, Sociology
P5	Zimbabwe	Programme coordinator, sexual and reproductive rights in Africa
P6	Ethiopia / Nigeria	Programme coordinator, public health, demography
P7	South Africa	Youth and gender activist
P8	Zimbabwe	Researcher and activist, medicine and public health
P9	Nigeria	Researcher, medicine and public health
P10	Niger	Programme director, business administration
P11	Gambia	Director, global health and public health
P12	South Africa	Researcher, gender studies
P13	Uganda	Researcher, human rights and psychology
P14	South Africa / Kenya	Researcher, development studies
P15	South Africa	Researcher, psychology and public health

The consultation workshop involved a series of iterative discussions exploring the drivers of imbalance and possible solutions, and reaching a shared understanding. These discussions have shaped our interpretation of the results and their implications, and a summary of the discussion is integrated into the paper.

The broader research project was approved by the Biomedical Research Ethics Committee of the University of the Western Cape on 15 November 2022 (BM22/9/2).

### Data synthesis

The data analysis was done sequentially. The data from the scoping review were analysed first using descriptive quantitative analysis. We employed pie charts, stacked bar plots, and bar graphs to present and visualise the data. Thematic analysis was then applied to analyse data from the expert consultations. Quality appraisal of the studies or assessment of the strength of the evidence in the studies was not done, as this is not a feature of scoping reviews and is not part of our research aim.

### Reflexivity

We are a team of multidisciplinary researchers based in South Africa, Benin, and India, with between 10 and 40 years of experience working in the field, advancing gender and SRHR nationally, regionally and globally. Those based in South Africa work for a public university that primarily relies on external grants to undertake research. We acknowledge the privilege we have to be able to undertake this research and the relative financial independence to ask critical questions about our field. Throughout the research process, we were mindful of our positionality and attentive to the contextual and power dynamics inherent in such work. We recognise the importance of mobilising knowledge from experts across a range of sectors, including academia, civil society, and activism, as well as programmes, to enrich our understanding and interpretation. In addition, we regularly consulted and debriefed with the Gender Transformation for Africa project’s steering committee, which includes representation from research projects across six countries in West, Eastern and Southern Africa, to ensure a reflexive and inclusive approach throughout the research process.

## Results

### Literature scope

The initial search generated 295 references, all written in English. Following the absence of French publications, we conducted another search on PubMed and Scopus using the French equivalents of the English search strings. This search produced 11 results, none of which met the eligibility criteria. Reviews were omitted from the study due to the study’s focus on primary research, and to take stock of how research applying gender approaches to SRH is operationalised and assessed.

**Figure 1.**
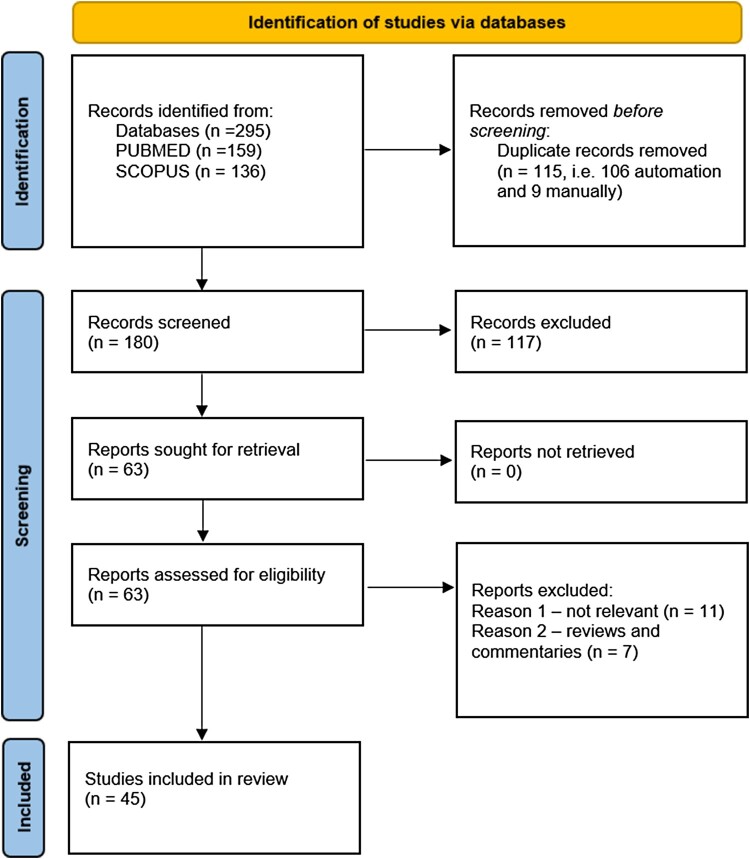
PRISMA flow chart

Forty-five references, which met the eligibility criteria, were considered for the synthesis (Figure 1).

### Distribution of articles across geographic sub-regions

**Figure 2.**
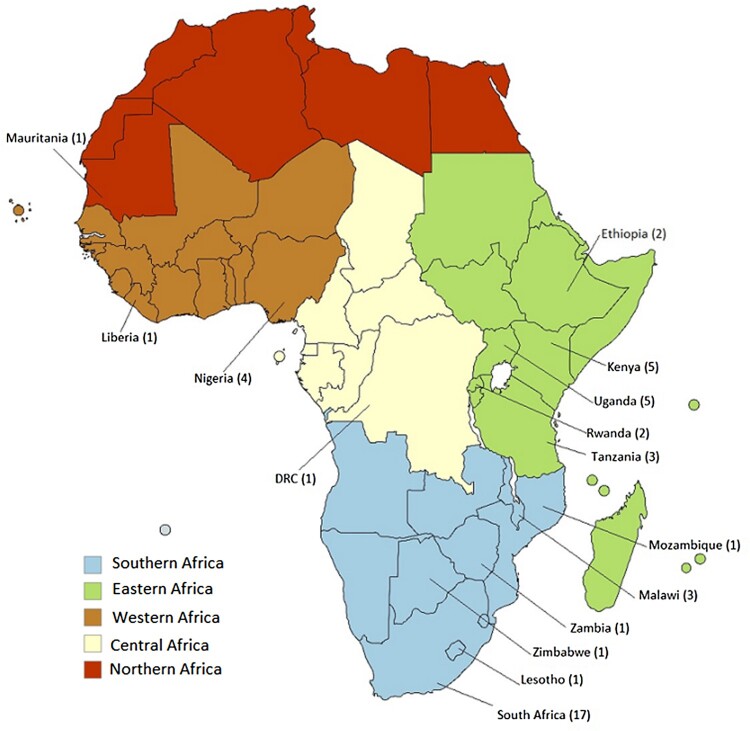
Distribution of 45 articles on gender and sexual and reproductive health across African regions from 2012 to 2022

With respect to the study setting, the papers were situated across 15 countries. Nearly half of the papers (22, 48.9%) were situated in Southern Africa, followed by 17 (37.8%) papers in Eastern Africa. The remaining papers focused on the Western Africa (5, 11.1%) and Northern Africa (1, 2.1%) regions. Among central African countries, only the DRC was included in a paper with a multi-country focus (Figure 2).

### Distribution of articles by study location and authorship

**Figure 3.**
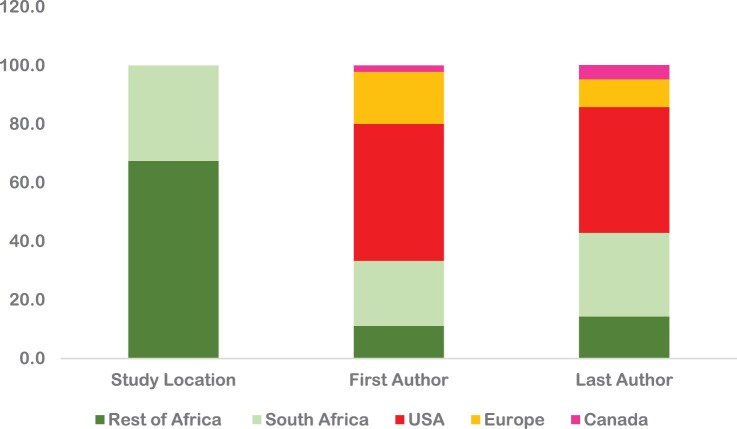
Distribution of 45 articles on gender and sexual and reproductive health across Africa from 2012 to 2022 by study location and authorship

The distribution of articles by study location starkly contrasts the country distribution of first and last authors; 67% of the first authors and 57% of the last authors were not based in Africa (Figure 3).

Authors from the United States (USA) are the most represented as first authors (46.7%). European authors account for one-fifth of the first authors (17.8%). From Africa, South Africa is the most represented (22.2%), which is double the percentage of authors from the rest of Africa (11.1%). Similar patterns prevail with respect to the distribution of last authors with authors from USA dominating (42.9%). European last authors feature in 9.5% of the papers. South African authors remain in the lead in last authorship roles (28.6%) among those on the continent, again double the percentage for their fellow authors from the rest of the continent (14.3%). Ten of the 45 articles had no local authors.

With respect to the South Africa-based authorship, out of 45 papers, 10 had South African first-authors, and 12 had South African last authors, with six having South African authors listed as both first and last authors. Among the ten papers with South African first authors, half of them were affiliated to Medical Research Council-South Africa. Of the 10 papers with South Africa-based first authors, three were conducted in Uganda,^43^ Rwanda,^52^ and Malawi,^41^ with the remaining seven located in South Africa. Looking specifically at the 12 papers last-authored by South African researchers, two were set in Uganda^43^ and Lesotho,^80^ and the rest were set in South Africa.

With regard to lead authorship among Africans who are not South African, among 45 papers, only five papers had non-South African African authors as first authors and six as last authors. The countries represented among the first authors included Nigeria (2), Tanzania (1), Senegal (1), and Kenya (1). Interestingly, only one of these first authors (from Senegal) conducted a study in a different setting (Mauritania). The countries represented by the last authors included Zimbabwe, Tanzania, Nigeria, Uganda, Ethiopia and Kenya, each represented by one paper. Each of these last authors conducted the study in their respective countries. Two papers had Tanzanian or Nigerian authors listed as both first and last authors.^79,81^

### Distribution of articles by authors’ affiliation

**Figure 4.**
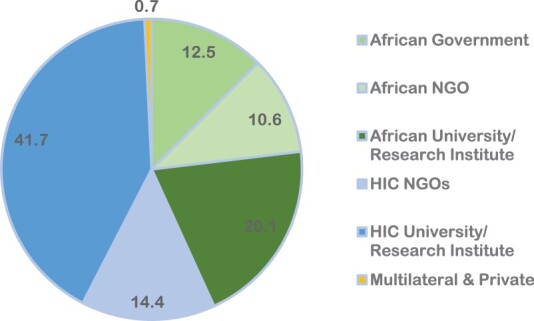
Distribution of 45 articles on gender and sexual and reproductive health across Africa from 2012 to 2022 by authors’ affiliation

With respect to the first organisational affiliation for the 264 authors included in the analysis, nearly three-fifths of authors were located in organisations in high-income countries, either at universities/research institutes (41.7%) or NGOs (14.4%). The remaining two-fifths of the authors were based in African institutions: African universities or research centres (20.1%), African governments (12.5%), and African NGOs (10.6%). Multilateral and private sources make up a fraction (0.7%) of the total (Figure 4).

**Figure 5.**
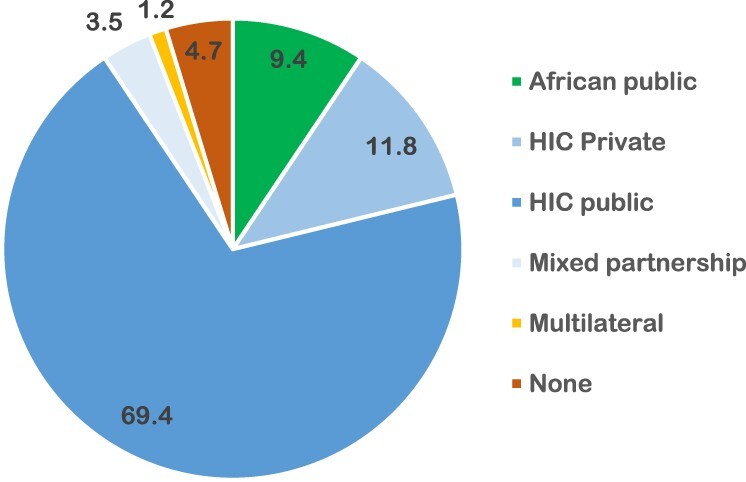
Distribution of 45 articles on gender and sexual and reproductive health across Africa from 2012 to 2022 by funding source

Our review highlighted the unequal access to external funding by in-country researchers and insufficient local funding. Reviewing the 85 reported funding sources shows a skewed distribution, with eight of the ten funders in high-income countries (HIC) (69.4% public and 11.8% private). In contrast, African funders represent only 9.4% of the funding. The remaining funding sources were multilateral (1.2%) and mixed (3.5%), with a few funding sources not declared (4.7%). The main HIC funding sources include the USA (36.5%), UK (14.1%), Canada (8.2%), and Sweden (5.9%). A closer look at the African sources of funding reveals that all eight funding sources reported in five papers were public institutions from South Africa, namely the Medical Research Council (5), National Research Fund (1), and the University of the Witwatersrand (2). In all these instances, the African funding sources were not sole funders, as they were joined by public and private funders from HIC. Of the five papers funded by South African sources, three had both the first and last authors based in South Africa. In the remaining two cases, South African authors held the last authorship role, while authors based in the USA occupied the first authorship positions (Figure 5).

### Consultation workshop: making sense of the imbalances

The experts at the consultation workshop concurred with the overall and distinct patterns evident in the findings regarding the distribution of research on sexual and reproductive health across sub-regions in Africa employing gender approaches, authorship affiliations, and funding sources. They further identified a diverse set of factors contributing to the imbalance, operating at multiple levels: global, national, institutional, and individual.

Participants highlighted various factors affecting the generally low number of publications and regional differences in African leadership authorship positions. As depicted in Figure 7, participants made references to structural drivers at national and institutional, and at global and regional levels. Furthermore, a set of drivers relates to the enduring presence of colonial legacies, which sustains the epistemic marginalisation of Global South researchers. This includes the dominance of the English language in global academic publishing. The lack of investment by African governments and funders in research on gender approaches in the health sector was flagged as a major concern, resulting in the reliance on external funding sources. The dominant biomedical orientation of research projects has further contributed to the limited consideration of gender and other social determinants.

**Figure 6.**
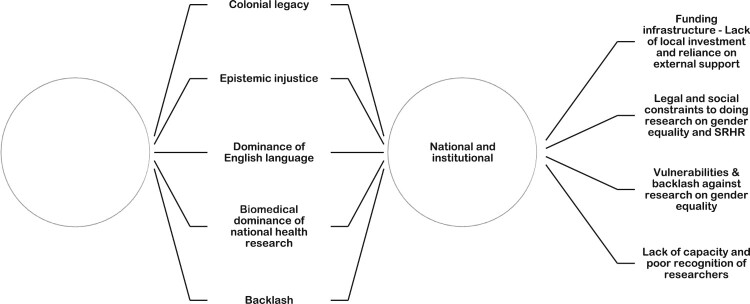
Drivers of imbalances in authorship, geographic and institutional contexts, and funding sources Source: From a consultation workshop with 15 experts across Africa ^84^

Another set of factors was more inward-looking and highlighted local restrictions and associated vulnerabilities. These include tensions between the transformative aspirations of research in gender equality and SRHR and the patriarchal and conservative contextual realities across many sub-Saharan African countries, especially with regard to legal and policy frameworks, and sociocultural and religious norms. Researchers reiterated the daunting nature of conducting research in this field, with security issues due to unrest or terrorism in certain parts of the continent. Researchers noted using alternative or less contentious language suited to their context to discuss sensitive issues (e.g. condom use, safe sex). This local self-censorship and the resulting use of alternative language may contribute to the relatively low number of articles picked up in the review, since these terms do not feature in our search strategies. The role and contribution of civil society and rights-based organisations in opening up space and shifting social structures that reproduce gender inequality were highlighted as critically important. Pockets of innovation by researchers in the region who navigate constraining local contexts to advance the field were also acknowledged (Figure 6).^84^

**Figure 7.**
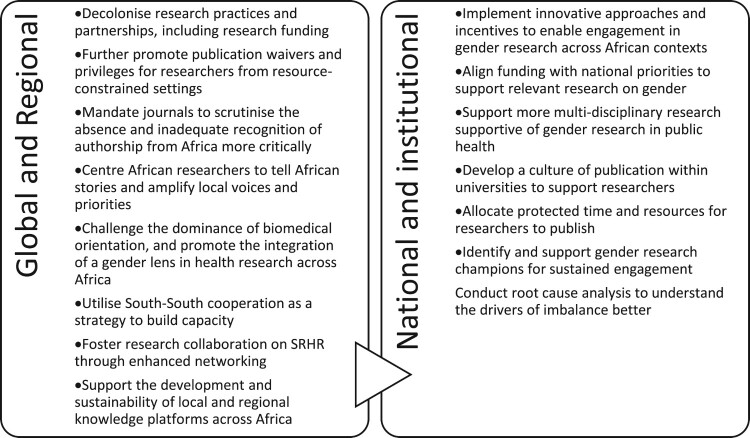
Strategies to address the reported imbalances in authorship, geographic and institutional contexts, and funding sources Source: From a consultation workshop with 15 experts across Africa ^84^

Participants underscored a general lack of local investment and support to develop the capacity of research institutions and researchers, which does not align with local and external expectations to publish, write, and compete for grants in order to improve participation in knowledge production and output at the global level. Participants shared experiences of being unable to write publications despite having generated data, due to a lack of time and resources. Academic staff face particularly heavy teaching loads due to the imperative for universities to increase enrolment and throughput. Against the above background, participants put forward various strategies to shift the existing imbalance (Figure 7). These include global and regional interventions that tackle systemic imbalances and their root causes, such as changes in funding, publishing, research priorities, and partnerships, as well as national and local measures that foster a supportive research ecosystem through increased funding and greater local ownership.

## Discussion

Our focused analysis of the scoping review examined patterns of leadership in authorship in SRH research across Africa integrating gender approaches. Our analysis revealed stark disparities in the distribution of research settings, lead authorship representation and funding sources among peer-reviewed articles. Of the 45 reviewed articles, 49% were situated in Southern Africa, 38% in Eastern Africa, and 13% in Western Africa. Extreme disparities prevail in the distribution of authors by country of residence, 67% of the first authors and 57% of the last authors were not based in Africa. One-fifth of the papers feature no local authors. Of the total reviewed articles, South African authors make up 22% of the first authors and 29% of the last authors, which is double the proportion for authors from the rest of Africa. Under 10% of the funding came from Africa, exclusively from South Africa, with the rest originating from high-income countries. Consultation with African gender and health experts helped to contextualise these findings, highlighting the local and global factors drivers of the imbalance.

### Regional biases

The review brings to light the imbalance in the regional distribution of papers across Africa, wherein fewer articles came out of Western and Northern Africa compared to Southern and Eastern Africa. This disparity is primarily attributed to the hegemony of the English language across global knowledge platforms, which disadvantages non-Anglophone speaking regions/countries. Further, the predominant use of English also affects the consequent uneven distribution of resources and the strengthening of the capacity of institutions in these settings. Consistent with the views of the consultation workshop participants, prior reviews and analyses have flagged the problematic dominant influence of the English language in academic knowledge production.^28,85^ A bibliometric analysis of primary research publications from low- and middle-income countries (LMIC) in nine prominent medical and global health journals during 2014–2016 found that 26.2% of the 416 Africa-based publications were from the East Africa sub-region, which the authors attribute to the legacy of the English language and the relative competitive advantage built over a period due to such legacy, including research infrastructure. The authors urged a more intentional approach to address the systematic overlooking of other language regions in Africa.^86^

Mattison et al. in their global mapping of research on SRHR found a higher concentration of co-author networks in South Africa and Sierra Leone.^35^ South Africa’s lead in the number of research outputs and authorship, compared to other countries in the continent, resonates with our review.^35^ This can be attributed to South Africa’s relatively higher governmental investment into public universities and research entities, even if this has come under strain more recently. While Sierra Leone does not feature in our review, its Anglophone tradition reinforces the general argument regarding English language dominance. The sub-regional and language region disparities flagged by the review demonstrate the need to go beyond assumptions of commonality or homogeneity across such a diverse and large continent.

### Restrictive local settings

Researching gender equality and SRHR issues represents interrogating and challenging issues that go against the grain of culture, tradition, law, and patriarchy and pose numerous intrinsic and extrinsic challenges to researchers and research institutions regarding safety, social capital, and career and professional progression. As much as there is progress to celebrate in other African contexts, SRH issues like abortion are criminalised, SRH services are shrouded with stigma and gender-based violence in many contexts remains culturally condoned.^15,22,87^

In addition to these challenges specific to gender and SRH research, views of participants from the consultation align with several studies^33,88^ that identified a broad spectrum of interlinked factors undermining research leadership in the Global South. The first set of constraints is internal to the local context, and encompasses issues related to the researcher (i.e. competing priorities, poor incentive arrangements, and lack of mentorship) and the researcher’s home institution (i.e. unfavourable policies and processes, poor collaboration, and disabling research ecosystems that impose ethical, technological, capacity and security constraints). Constraints also involve structural-level factors (i.e. lack of local funding and support; political unrest and undue political pressure that influences the safety and credibility of researchers).

### Persistent bias favouring high-income country researchers

The predominance of high-income country authorship in SRH research applying gender approaches across Africa reflects historic investment in research capabilities and educational sectors in the global North vs disinvestment in the global South. Researchers from the Global North, until recently, have enjoyed relatively supportive research environments, career structures framed around competitive grants and authorship, and relatively secure livelihoods^88^ notwithstanding the ever increasing market-oriented university and research environments. Biases favouring participation from high-income countries (HIC) in research partnerships recur if not actively monitored and continuously addressed.^89,90^ A study that examines five major databases focusing on decolonising global health and global health partnerships found that 70% of the studies have only HIC-affiliated authors.^91^ A bibliometric analysis of primary research publications from LMIC in nine prominent medical and global health journals revealed that 28.8% of publications have no local author.^86^ These findings are consistent with our review, which showed that 20.0% of the papers lacked a local author.

The governance and management of research partnerships with Global North actors, and specifically the allocation of resources and roles, continue to shape knowledge production, translation and dissemination practices, including authorship. If the need for solidarity and support to overcome constraints faced by Global South partners is not recognised, it can further undermine the capacity strengthening of research leadership in the Global South.^33,90^ Research partnerships that leave the privileges of local elites unchecked are also problematic^92^ as highlighted in the regional differences in our results, with some sub-regions becoming hubs of authorship and funding networks, while others remain largely overlooked.

### Poor local investment and unequal access to external funding

Our review revealed unequal access to external funding for in-country researchers and insufficient local funding, with only one-tenth of the funding originating in Africa. This aligns with findings that establish funding from HICs as the primary source of funding for research in LMIC.^93^ Another study measuring authorship and funding of multi-country research partnerships in the area of HIV and AIDS in Africa found that while the funding source for 43 of the 77 published trials was HICs, only five had part or full funding from Africa.^94^ The general lack of research funding by governments in sub-Saharan Africa persists, despite recurrent calls for governments to live up to their pledges and responsibilities,^95^ even amidst increasing budget constraints.

A related finding of our review is the stronger representation of South-Africa-based researchers in a lead author role, compared to their counterparts in the rest of Africa, which coincides with stronger participation of South African public institutions in funding the research, even if co-financed with external funders. Other studies corroborate this link between local funding and lead authorship representation. A study examining first-authorship for randomised controlled trials among LMIC researchers over two decades identified a pattern whereby the likelihood of researchers from LMIC to feature as first authors was higher when the funding source is from LMIC, and vice versa.^96^

### Call to action

While the paper maps the landscape of research on this topic across the continent, it also serves as a real call to action, as warranted by the findings from the review and other related studies.

#### Multipronged and sustained approach

Addressing this persistent dynamic in research partnerships focused on applying gender approaches to sexual and reproductive health knowledge production and dissemination, requires a multipronged and sustained approach from all parties concerned. Similar studies to the review as well as the Consultation Workshop in the present study called for reckoning with the multifaceted internal and external constraints as a foundation for proper intervention.^25,33,97,98^ The need for overhauling the inequitable arrangements in global research partnerships has been taken up by regional and global initiatives, including The Africa Charter for Transformative Research Collaborations,^90^ and The Global Code of Conduct for Equitable Research Partnerships.^99^

#### Tracking progress

A systematic and comprehensive documentation of initiatives aimed at reversing the unequal power dynamics and privileges that undermine leadership development in knowledge production in Africa at various levels can provide valuable insights for addressing the issue in the future.

#### Disrupting the cycle of inequality

Strengthening research leadership capacity and ecosystems primarily involves creating space to set and pursue locally relevant research priorities and imagining models of resource distribution that nurture distributed leadership and exchange, foster partnerships with southern partners, and increase various funding streams.^25,33,35,90,97,98^ This includes encouraging further collaboration with the social sciences, where gender analysis originated.

#### Reflexivity

Reflexivity about power dynamics in international partnerships is important for all fields, particularly for those engaged in understanding and addressing social inequality.^97^ None of the articles in our focused analysis of the scoping review were reflexive about the underlying power dynamic and contributing factors in their engagement with SRHR issues. Such a lack of reflexivity is also observed in papers that explore power imbalance including those that call for decolonising global health.^100^

#### Domestic financing

A meaningful shift could also come from regional and national bodies investing in gender equality research in health, fostering local knowledge platforms, and strengthening research leadership capacity. Addressing domestic financing gaps has become even more urgent in light of recent funding cuts. In response, African states, under the leadership of the Africa CDC, have proposed a three-pronged strategy focused on domestic resource mobilisation, innovative financing, and blended finance.^8^

### Limitations

The review is limited to two databases and journals indexed under these, even if they are the most widely-used ones for public health. Limiting the search to these two databases reinforced the dominance of the English language in academic knowledge production. It may be that African social scientists who specialise in gender studies do not publish in public health journals. The review has not included grey literature, which also may have more African lead authorship, particularly from civil society. This paper also focuses specifically on research leadership and research ecosystems (authorship, funding, research), not content. While we have used the first institutional affiliation of authors as a proxy for the country of residence of authors, the study cannot determine with certainty the origin of the authors or the contribution of the African diaspora. The limitations to sufficiently account for the nuances in the profile of authors have also been reported by other authorship analysis papers.^34,101^

## Conclusion

Local ownership and leadership of SRH research grounded in gender approaches, undertaken through equitable research partnerships, is required in diverse contexts in Africa for impact over the long run. Realising this requires addressing the drivers of the imbalance at multiple levels. Structurally, it is imperative to challenge the coloniality embedded in global health research partnerships and funding allocations. Furthermore, journals in high-income countries should uphold equitable authorship practices in publishing research from international partnerships.^97^

As conservative campaigns against gender inequality across Africa intensify, misguiding audiences by conflating Western bias with efforts to support gender equality, more critical reflections about the nature of the scholarship and its long-term sustained contribution to local knowledge translation and progressive change warrant attention.

## Supplementary Material

Supplementary data charting table

## Appendix

### Preferred Reporting Items for Systematic reviews and Meta-Analyses extension for Scoping Reviews (PRISMA-ScR) Checklist

**Table:**

SECTION	ITEM	PRISMA-ScR CHECKLIST ITEM	REPORTED ON PAGE #
**TITLE**			
Title	1	Identify the report as a scoping review.	1
**ABSTRACT**			
Structured summary	2	Provide a structured summary that includes (as applicable): background, objectives, eligibility criteria, sources of evidence, charting methods, results, and conclusions that relate to the review questions and objectives.	2
**INTRODUCTION**			
Rationale	3	Describe the rationale for the review in the context of what is already known. Explain why the review questions/objectives lend themselves to a scoping review approach.	2 and 6
Objectives	4	Provide an explicit statement of the questions and objectives being addressed with reference to their key elements (e.g. population or participants, concepts, and context) or other relevant key elements used to conceptualise the review questions and/or objectives.	2 and 6
**METHODS**			
Protocol and registration	5	Indicate whether a review protocol exists; state if and where it can be accessed (e.g. a Web address); and if available, provide registration information, including the registration number.	8
Eligibility criteria	6	Specify characteristics of the sources of evidence used as eligibility criteria (e.g. years considered, language, and publication status), and provide a rationale.	8
Information sources*	7	Describe all information sources in the search (e.g. databases with dates of coverage and contact with authors to identify additional sources), as well as the date the most recent search was executed.	2 and 7
Search	8	Present the full electronic search strategy for at least 1 database, including any limits used, such that it could be repeated.	41
Selection of sources of evidence†	9	State the process for selecting sources of evidence (i.e. screening and eligibility) included in the scoping review.	8
Data charting process‡	10	Describe the methods of charting data from the included sources of evidence (e.g. calibrated forms or forms that have been tested by the team before their use, and whether data charting was done independently or in duplicate) and any processes for obtaining and confirming data from investigators.	9
Data items	11	List and define all variables for which data were sought and any assumptions and simplifications made.	9
Critical appraisal of individual sources of evidence§	12	If done, provide a rationale for conducting a critical appraisal of included sources of evidence; describe the methods used and how this information was used in any data synthesis (if appropriate).	Click here to enter text.
Synthesis of results	13	Describe the methods of handling and summarising the data that were charted.	11
**RESULTS**			
Selection of sources of evidence	14	Give numbers of sources of evidence screened, assessed for eligibility, and included in the review, with reasons for exclusions at each stage, ideally using a flow diagram.	40
Characteristics of sources of evidence	15	For each source of evidence, present characteristics for which data were charted and provide the citations.	41–46
Critical appraisal within sources of evidence	16	If done, present data on critical appraisal of included sources of evidence (see item 12).	Click here to enter text.
Results of individual sources of evidence	17	For each included source of evidence, present the relevant data that were charted that relate to the review questions and objectives.	41–46
Synthesis of results	18	Summarise and/or present the charting results as they relate to the review questions and objectives.	12–17
**DISCUSSION**			
Summary of evidence	19	Summarise the main results (including an overview of concepts, themes, and types of evidence available), link to the review questions and objectives, and consider the relevance to key groups.	21
Limitations	20	Discuss the limitations of the scoping review process.	29
Conclusions	21	Provide a general interpretation of the results with respect to the review questions and objectives, as well as potential implications and/or next steps.	30
FUNDING			
	22	Describe sources of funding for the included sources of evidence, as well as sources of funding for the scoping review. Describe the role of the funders of the scoping review	31

JBI = Joanna Briggs Institute; PRISMA-ScR = Preferred Reporting Items for Systematic reviews and Meta-Analyses extension for Scoping Reviews.

*Where *sources of evidence* (see second footnote) are compiled from, such as bibliographic databases, social media platforms, and Web sites.

^†^A more inclusive/heterogeneous term used to account for the different types of evidence or data sources (e.g. quantitative and/or qualitative research, expert opinion, and policy documents) that may be eligible in a scoping review as opposed to only studies. This is not to be confused with *information sources* (see first footnote).

^‡^The frameworks by Arksey and O’Malley (6) and Levac and colleagues (7) and the JBI guidance (4, 5) refer to the process of data extraction in a scoping review as data charting.

^§^The process of systematically examining research evidence to assess its validity, results, and relevance before using it to inform a decision. This term is used for items 12 and 19 instead of “risk of bias” (which is more applicable to systematic reviews of interventions) to include and acknowledge the various sources of evidence that may be used in a scoping review (e.g. quantitative and/or qualitative research, expert opinion, and policy document).

*From:* Tricco AC, Lillie E, Zarin W, O'Brien KK, Colquhoun H, Levac D, et al. PRISMA Extension for Scoping Reviews (PRISMAScR): Checklist and Explanation. Ann Intern Med. 2018;169:467–473. doi: 10.7326/M18-0850.
