# Supplementary material for: Imbalances in authorship, geographic and institutional contexts, and funding sources in research on gender approaches to sexual and reproductive health in Africa: a scoping review
Source: Sex Reprod Health Matters. 2026 Jan 16;33(1):2616137. doi: 10.1080/26410397.2026.2616137 (PMC13094261; doi:10.1080/26410397.2026.2616137)
Supplement: Supplementary data charting table [file ZRHM_A_2616137_SM3369.docx]

Supplementary data charting table of articles on gender transformative approaches to sexual, reproductive and maternal health in sub-Saharan Africa (2012-2022)

A more comprehensive data charting table on the content of the reviewed paper is included in the main scoping review by George et al. (2025), available at <https://www.ajrh.info/index.php/ajrh/article/view/5693/2271>

| **S No** | **Author** | **Title of the paper** | **First author's country** | **Last author's country** | **Total number of local authors** | **Total number of authors** | **Each author's first organisational affiliation** | **Funding source/s** | **Year** | **Countries study is situated** |
| --- | --- | --- | --- | --- | --- | --- | --- | --- | --- | --- |
|  | (Doyle *et al.*, 2014) | Transforming gender roles in domestic and caregiving work: preliminary findings from engaging fathers in maternal, newborn, and child health in Rwanda | USA | USA | 1 | 4 | Promundo-US. NW, Washington Promundo-US.NW, Washington, DC  MenCare+, Rwanda Men’s Resource Center Promundo-US, NW, Washington, | Dutch Ministry for Foreign Affairs | 2014 | Rwanda |
|  | (Gibbs, Jewkes and Sikweyiya, 2018) | “I Tried to Resist and Avoid Bad Friends”: The Role of Social Contexts in Shaping the Transformation of Masculinities in a Gender Transformative and Livelihood Strengthening Intervention in South Africa | South Africa | South Africa | 3 | 3 | Gender and Health Research Unit, South African Medical Research Council Gender and Health Research Unit, South African Medical Research Council Gender and Health Research Unit, South African Medical Research Council | Swedish International Development Agency (Sida), Norwegian Agency for Development Cooperation (Norad), the Joint Gender Fund (South Africa), and the Medical Research Council of South Africa (SAMRC) | 2018 | South Africa |
|  | (Kedde *et al.*, 2018) | MenCare+ in South Africa: findings from a gender transformative young men’s group education on sexual and reproductive health and rights | Netherlands | South Africa | 2 | 4 | Rutgers, Utrecht, The Netherlands Mosaic, Cape Town, South Africa Sonke Gender Justice, Cape Town, South Africa Sonke Gender Justice, Cape Town, South Africa | Ministry of Foreign Affairs-Netherlands | 2018 | South Africa |
|  | (Mkandawire, Hendriks and Mkandawire-Vahlmu, 2018) | A gender assessment of Malawi’s National Nutrition Policy and Strategic Plan 2007–2012 | South Africa | USA | 0 | 3 | Department of Agricultural Economics, Agricultural Extension and Rural Development, and research Assistant, Institute for Food, Nutrition and Well-being at the University of Pretoria.  Institute for food, Nutrition and Wellbeing in the Department of Agricultural Economics, Extension and Rural Development, University of Pretoria. College of Nursing, University of Wisconsin–Milwaukee | USAID-Feed the Future initiative’s Innovation Lab for Food Security Policy | 2018 | Malawi |
|  | (Seff *et al.*, 2023) | A Family‑Focused, Sibling‑Synchronous Intervention in Borno State, Nigeria: Exploring the Impact on Family Functioning and Household Gender Roles | USA | USA | 1 | 10 | Brown School of Social Work, Washington University  Mercy Corps Nigeria, Maiduguri, Borno State, Nigeria. Brown School of Social Work, Washington University  Brown School of Social Work, Washington University  Brown School of Social Work, Washington University  Women’s Refugee Commission, New York, NY, USA Women’s Refugee Commission, New York, NY, USA Mercy Corps Nigeria, Maiduguri, Borno State, Nigeria. Women’s Refugee Commission, New York, NY, USA Brown School of Social Work, Washington University | Government of Canada- Global Affairs Canada | 2022 | Nigeria |
|  | (Stern *et al.*, 2015) | Lessons learned from engaging men in sexual and reproductive health as clients, partners and advocates of change in the Hoima district of Uganda | South Africa | South Africa | 0 | 4 | Women’s Health Research Unit, University of Cape Town, Cape Town, South Africa;  Sonke Gender Justice, Cape Town, South Africa; Sonke Gender Justice, Cape Town, South Africa; Centre for Social Science Research, University of Cape Town, Cape Town, South Africa | Swedish Association for Sexuality Education (RFSU). | 2015 | Uganda |
|  | (Gibbs *et al.*, 2020) | Constructing, reproducing and challenging masculinities in a participatory intervention in urban informal settlements in South Africa | South Africa | South Africa | 4 | 5 | South African Medical Research Council, Gender and Health Research Unit, Pretoria, South Africa; International Alert, London, UK;  Project Empower, Durban, South Africa; South African Medical Research Council, Gender and Health Research Unit, Pretoria, South Africa; South African Medical Research Council, Gender and Health Research Unit, Pretoria, South Africa; | DFID | 2020 | South Africa |
|  | (Hampanda *et al.*, 2020) | Support or control? Qualitative interviews with Zambian women on male partner involvement in HIV care during and after pregnancy | USA | USA | 2 | 7 | Department of Obstetrics and Gynaecology, School of Medicine, University of Colorado Anschutz Medical Campus, Aurora, Colorado, United States of America; Department of Health Promotion and Education, School of Public Health, University of Zambia, Lusaka, Zambia; Department of Obstetrics and Gynaecology, Women and Newborn Hospital, University Teaching Hospitals, Lusaka, Zambia; Department of Health Behaviour, Gillings School of Global Public Health, University of North Carolina, Chapel Hill, North Carolina, United States of America; Department of Health Care Organization and Policy, School of Public Health, University of Alabama at Birmingham Alabama, United States of America; Department of Health Behavior and Biological Sciences, Center for Sexuality and Health Disparities, School of Nursing, University of Michigan, Ann Arbor, Michigan, United States of America; Center for Global Health, Colorado School of Public Health, University of Colorado Anschutz Medical Campus, Aurora, Colorado, United States of America | National Institute of Mental Health, NIH, USA | 2020 | Zambia |
|  | (Koris *et al.*, 2022) | Opportunities and challenges in preventing violence against adolescent girls through gender transformative, whole‑family support programming in Northeast Nigeria | USA | USA | 4 | 9 | Women’s Refugee Commission, 15 W. 37th St, New York, NY 10018, USA. Mercy Corps Nigeria, 35 Patrick Bokkor Crescent, Jabi, Utako District, Abuja, Nigeria. Mercy Corps Nigeria, 35 Patrick Bokkor Crescent, Jabi, Utako District, Abuja, Nigeria. Women’s Refugee Commission, 15 W. 37th St, New York, NY 10018, USA. Mercy Corps Nigeria, 35 Patrick Bokkor Crescent, Jabi, Utako District, Abuja, Nigeria. Mercy Corps Nigeria, 35 Patrick Bokkor Crescent, Jabi, Utako District, Abuja, Nigeria. Brown School at Washington University in St. Louis, Campus Box 1196, 1 Brookings Drive, St. Louis, MO 63130, USA Women’s Refugee Commission, 15 W. 37th St, New York, NY 10018, USA. Brown School at Washington University in St. Louis, Campus Box 1196, 1 Brookings Drive, St. Louis, MO 63130, USA | Government of Canada through Global Affairs Canada | 2022 | Nigeria |
|  | (Harrington *et al.*, 2016) | Gendered power dynamics and women’s negotiation of family planning in a high HIV prevalence setting: a qualitative study of couples in western Kenya | USA | USA | 2 | 6 | Department of Obstetrics and Gynaecology, Oregon Health and Science University, Portland, USA; Department of Social and Behavioral Sciences, University of California, San Francisco, USA; Institute for Global Health, University of Southern California Keck School of Medicine, Los Angeles, USA; Research, Care, and Training Program, Center for Microbiology Research, Kenya Medical Research Institute, Kisumu, Kenya; Research, Care, and Training Program, Center for Microbiology Research, Kenya Medical Research Institute, Kisumu, Kenya; Department of Obstetrics, Gynecology and Reproductive Sciences, San Francisco General Hospital, University of California, San Francisco, USA | Hellman Family Foundation The National Institutes of Health (NIH), UCSF-Gladstone Institute of Virology & Immunology Center for AIDS Research | 2016 | Kenya |
|  | (Harvey *et al.*, 2021) | A cluster randomised controlled trial to evaluate the impact of a gender transformative intervention on intimate partner violence against women in newly formed neighbourhood groups in Tanzania | UK | UK | 2 | 11 | Global Health and Development, Faculty of Public Health and Policy, London School of Hygiene & Tropical Medicine, London, UK; Global Health and Development, Faculty of Public Health and Policy, London School of Hygiene & Tropical Medicine, London, UK; Sexual and Reproductive Health, National Institute for Medical Research Mwanza Research Centre, Mwanza, Mwanza, Tanzania; Infectious Disease Epidemiology, Faculty of Epidemiology and Population Health, London School of Hygiene & Tropical Medicine, London, UK Mwanza Intervention Trials Unit, National Institute for Medical Research Mwanza Research Centre, Mwanza, Tanzania; Mwanza Intervention Trials Unit, National Institute for Medical Research Mwanza Research Centre, Mwanza, Tanzania; Mwanza Intervention Trials Unit, National Institute for Medical Research Mwanza Research Centre, Mwanza, Tanzania; Mwanza Intervention Trials Unit, National Institute for Medical Research Mwanza Research Centre, Mwanza, Tanzania; Global Health and Development, Faculty of Public Health and Policy, London School of Hygiene & Tropical Medicine, London, UK; Global Health and Development, Faculty of Public Health and Policy, London School of Hygiene & Tropical Medicine, London, UK; Mwanza Intervention Trials Unit, National Institute for Medical Research Mwanza Research Centre, Mwanza, Tanzania; | UK Aid from the Department for International development | 2021 | Tanzania |
|  | (Chzhen *et al.*, 2021) | Impact of a cash plus intervention on gender attitudes among Tanzanian adolescents | Ireland | USA | 2 | 6 | Trinity college, Dublin; Erasmus University Medical Center, Rotterdam;  UNICEF, Dar es salaam; Tanzania Social Action Fund, Dodoma; Tanzania Social Action Fund, Dodoma; State University of New York | Oak foundation, UNICEF Tanzania, SIDA, UK Department for International Development and Irish Aid | 2021 | Tanzania |
|  | (Conroy, Ruark and Tan, 2020) | Re-conceptualising gender and power relations for sexual and reproductive health: contrasting narrative of tradition, unity, and rights | USA | USA | 0 | 3 | Center for AIDS Prevention Studies, University of California San Francisco, San Francisco; Department of Medicine, Brown University, Providence Center for AIDS Prevention Studies, University of California San Francisco, San Francisco; | US National Institutes of Health | 2020 | Malawi |
|  | (Closson *et al.*, 2019) | Measuring sexual relationship power equity among young women and young men south Africa: Implications for gender-transformative programming | Canada | Canada | 4 | 11 | Faculty of Health Sciences, Simon Fraser University, Burnaby, British Columbia, Canada,  Faculty of Health Sciences, University of the Witwatersrand, Johannesburg, South Africa; Maternal Adolescent and Child Health (MatCH) Research Unit (MRU), Faculty of Health Sciences, University of the Witwatersrand, Durban, South Africa; South African Medical Research Council, Cape Town, South Africa,  Faculty of Health Sciences, University of the Witwatersrand, Johannesburg, South Africa; Faculty of Health Sciences, Simon Fraser University, Burnaby, British Columbia, Canada,  Maternal Adolescent and Child Health (MatCH) Research Unit (MRU), Faculty of Health Sciences, University of the Witwatersrand, Durban, South Africa; Faculty of Health Sciences, University of the Witwatersrand, Johannesburg, South Africa; HIV Pathogenesis Programme and Africa Health Research Institute, University of KwaZulu-Natal, Durban, South Africa; Faculty of Health Sciences, Simon Fraser University, Burnaby, British Columbia, Canada,  Faculty of Health Sciences, Simon Fraser University, Burnaby, British Columbia, Canada, | Canadian Institutes for Health Research and the Canadian HIV Vaccine Initiative | 2019 | South Africa |
|  | (Dunkle *et al.*, 2020) | Effective prevention of intimate partner violence through couples training: a randomised controlled trial of Indashyikirwa in Rwanda | South Africa | USA | 0 | 4 | Gender and Health Research Unit, South African Medical Research Council, Tygerberg, Western Cape, South Africa; Gender Violence and Health Centre, London School of Hygiene & Tropical Medicine, UK; Department of Population, Family and Reproductive Health, Johns Hopkins University Bloomberg School of Public Health, Baltimore, Maryland, USA; Department of Population, Family and Reproductive Health, Johns Hopkins University Bloomberg School of Public Health, Baltimore, Maryland, USA; | UK Government’s Department for International Development (DFID) | 2020 | Rwanda |
|  | (Dworkin *et al.*, 2013) | Impact of a Gender-Transformative HIV and Anti-violence Program on Gender Ideologies and Masculinities in Two Rural, South African Communities | USA | South Africa | 2 | 4 | Department of Social and Behavioral Sciences, University of California at San Francisco, San Francisco, CA, USA;  Bixby Center for Global Reproductive Health, University of California at San Francisco, San Francisco, CA, USA; Centre for Infectious Disease Epidemiology and Research (CIDER), School of Public Health and Family Medicine, University of Cape Town, South Africa; Sonke Gender Justice, Cape Town, South Africa | National Institutes of Health,University of California, San Francisco- Gladstone Institute of Virology & Immunology Center for AIDS Research | 2012 | South Africa |
|  | (Mukumbang, 2021) | Leaving No Man Behind: How Differentiated Service Delivery Models Increase Men’s Engagement in HIV Care | South Africa | South Africa | 1 | 1 | South African Medical Research Council, Cape Town, South Africa. | Funding information not provided | 2020 | South Africa |
|  | (Gibbs *et al.*, 2015) | Reconstructing masculinity? A qualitative evaluation of the Stepping Stones and Creating futures interventions in urban informal settlements in South Africa | South Africa | South Africa | 4 | 4 | Health Economics and HIV and AIDS Research Division, University of KwaZulu-Natal, Durban; Gender and Health Research Unit, Medical Research Council, Pretoria Gender and Health Research Unit, Medical Research Council, Pretoria Health Economics and HIV and AIDS Research Division, University of KwaZulu-Natal, Durban; | SIDA and Norad, the Joint Gender Fund (South Africa) and the Medical Research Council, South Africa | 2015 | South Africa |
|  | (Ghanotakis *et al.*, 2017) | Evaluation of a male engagement intervention to transform gender norms and improve family planning and HIV service uptake in Kabale, Uganda | USA | Uganda | 2 | 9 | Elizabeth FHI Elizabeth Glaser Pediatric AIDS Foundation, Washington, DC, USA;  FHI 360, Durham, NC, USA FHI 360, Durham, NC, USA FHI 360, Durham, NC, USA FHI 360, Durham, NC, USA Elizabeth FHI Elizabeth Glaser Pediatric AIDS Foundation, Washington, DC, USA;  Elizabeth Glaser Pediatric AIDS Foundation, Kampala, Uganda Elizabeth FHI Elizabeth Glaser Pediatric AIDS Foundation, Washington, DC, USA;  Health Department, Kabale District Local Government, Uganda | USAID | 2017 | Uganda |
|  | (Mudi *et al.*, 2021) | Impact of home visits to pregnant women and their spouses on gender norms and dynamics in Bauchi State, Nigeria: Narratives from visited men and women | Nigeria | Canada | 5 | 10 | Bauchi State College of Nursing and Midwifery, Bauchi, Nigeria. Federation of Muslim Women Association of Nigeria (FOMWAN), Bauchi State, Nigeria. CIET/PRAM, Department of Family Medicine, McGill University, Montreal, Quebec, Canada. Centro de Investigación de Enfermedades Tropicales (CIET), Universidad Autónoma de Guerrero, Acapulco, Mexico. Centro de Investigación de Enfermedades Tropicales (CIET), Universidad Autónoma de Guerrero, Acapulco, Mexico. Federation of Muslim Women Association of Nigeria (FOMWAN), Bauchi State, Nigeria. Federation of Muslim Women Association of Nigeria (FOMWAN), Bauchi State, Nigeria. Bauchi State Ministry of Health, Bauchi, Nigeria. CIET/PRAM, Department of Family Medicine, McGill University, Montreal, Quebec, Canada. CIET/PRAM, Department of Family Medicine, McGill University, Montreal, Quebec, Canada. | Innovating for Maternal and Child Health in Africa, initiative, a partnership of Global Affairs Canada (GAC), the  Canadian Institutes of Health Research (CIHR) and Canada’s  International Development Research Centre (IDRC | 2021 | Nigeria |
|  | (Jacobs, George and De Jong, 2021) | Policy foundations for transformation: a gender analysis of adolescent health policy documents in South Africa | South Africa | South Africa | 3 | 3 | School of Public Health, University of the Western Cape, South Africa School of Public Health, University of the Western Cape, South Africa School of Public Health, University of the Western Cape, South Africa | South African Research Chair’s Initiative of the Department of Science and Technology and National Research Foundation of South Africa (Grant No 82769) and the South African Medical Research Council. The paper is part of the work undertaken by the Drivers Technical Working Group from Countdown 2030 funded by the Bill and Melinda Gates Foundation | 2021 | South Africa |
|  | (Decker *et al.*, 2021) | Gendered health, economic, social and safety impact of COVID-19 on adolescents and young adults in Nairobi, Kenya | USA | Belgium | 3 | 15 | Department of Population, Family and Reproductive Health, Johns Hopkins Bloomberg School of Public Health, Baltimore, MD, United States of America,  Department of Population, Family and Reproductive Health, Johns Hopkins Bloomberg School of Public Health, Baltimore, MD, United States of America,  International Centre for Reproductive Health-Kenya, Nairobi, Kenya,  Department of International Health, Johns Hopkins Bloomberg School of Public Health, Baltimore, MD, United States of America, Department of International Health, Johns Hopkins Bloomberg School of Public Health, Baltimore, MD, United States of America, Johns Hopkins Carey Business School, Baltimore, United States of America, Department of Population, Family and Reproductive Health, Johns Hopkins Bloomberg School of Public Health, Baltimore, MD, United States of America,  Department of Population, Family and Reproductive Health, Johns Hopkins Bloomberg School of Public Health, Baltimore, MD, United States of America,  Johns Hopkins Carey Business School, Baltimore, United States of America Department of Sociology, Gender and Development Studies, Kenyatta University, Nairobi, Kenya Department of Population, Family and Reproductive Health, Johns Hopkins Bloomberg School of Public Health, Baltimore, MD, United States of America,  Johns Hopkins School of Nursing, Baltimore, MD, United States of America Department of Population, Family and Reproductive Health, Johns Hopkins Bloomberg School of Public Health, Baltimore, MD, United States of America,  Department of Population, Family and Reproductive Health, Bill & Melinda Gates Institute for Population and Reproductive Health, Johns Hopkins Bloomberg School of Public Health, Baltimore, MD, United States of America International Centre for Reproductive Health-Kenya, Nairobi, Kenya, | Bill & Melinda Gates Foundation | 2021 | Kenya |
|  | (Pettifor *et al.*, 2018) | Community mobilization to modify harmful gender norms and reduce HIV risk: results from a community cluster randomized trial in South Africa | USA | South Africa | 9 | 13 | University of North Carolina University of the Witwatersrand Population Council- Washington University of North Carolina University of North Carolina  Sonke Gender Justice, Cape Town University of North Carolina Sonke Gender Justice, Cape Town University of the Witwatersrand  University of the Witwatersrand  University of the Witwatersrand  University of the Witwatersrand  University of the Witwatersrand | Carolina Population Center; Faculty of Health Sciences, University of the Witwatersrand and the Medical Research Council, South Africa; the Wellcome Trust, UK | 2018 | South Africa |
|  | (Sharma *et al.*, 2020) | Effectiveness of a culturally appropriate intervention to prevent intimate partner violence and HIV transmission among men, women, and couples in rural Ethiopia: Findings from a cluster-randomized controlled trial | USA | Ethiopia | 2 | 5 | Massachusetts Institute of Technology, Cambridge, United States of America; International Food Policy Research Institute, Washington, DC, United States of America; CARE, New York, United States of America; EngenderHealth, Addis Ababa, Ethiopia; Ethiopian Public Health Association, Addis Ababa, Ethiopia | anonymous donor, and the Fondation de France | 2020 | Ethiopia |
|  | (Sileo *et al.*, 2019) | Masculinity and engagement in HIV care among male fisherfolk on HIV treatment in Uganda | USA | USA | 5 | 10 | San Diego State University School of Public Health, San Diego, CA, USA; San Diego State University School of Public Health, San Diego, CA, USA; Mildmay Uganda, Kampala, Uganda; University of California San Diego, La Jolla, CA, USA University of California San Diego, La Jolla, CA, USA School of Public Health, Makerere University, Kampala, Uganda Mildmay Uganda, Kampala, Uganda; Mildmay Uganda, Kampala, Uganda; Mildmay Uganda, Kampala, Uganda; San Diego State University School of Public Health, San Diego, CA, USA; | GloCal Health Fellowship, National Institute of Health Fogarty International Center and the University of California Global Health Institute, Predoctoral Fellowship Award on Substance Abuse, HIV and Related Infections from the National Institute of Drug Abuse | 2019 | Uganda |
|  | (Sprague, Woollett and Hatcher, 2020) | Enhancing agency for health providers and pregnant women experiencing intimate partner violence in South Africa | USA | USA | 0 | 3 | School of Public Health, Faculty of Health Sciences, University of the Witwatersrand, Johannesburg, South Africa; School of Public Health, University of Nairobi, Kenya; | None declared | 2020 | South Africa |
|  | (Thuita *et al.*, 2021) | Fathers and grandmothers experiences participating in nutrition peer dialogue groups in Vihiga County, Kenya | Kenya | USA | 2 | 6 | USAID Advancing Nutrition/Department of Global Health,  Save the Children, Washington, DC, USA; Independent Consultant, Nairobi, Kenya; Department of Nutrition, Gillings School of Global Public Health, University of North Carolina at Chapel Hill, Chapel Hill, North Carolina, USA Department of Nutrition, Gillings School of Global Public Health, University of North Carolina at Chapel Hill, Chapel Hill, North Carolina, USA Department of Nutrition, Gillings School of Global Public Health, University of North Carolina at Chapel Hill, Chapel Hill, North Carolina, USA | National Institutes of Health, Grant/Award; United States Agency for International Development, Grant | 2021 | Kenya |
|  | (Treves-Kagan *et al.*, 2020) | Fostering gender equality and alternatives to violence: perspectives on a gender-transformative community mobilisation programme in rural South Africa | USA | South Africa | 6 | 9 | Department of Health Behavior, University of North Carolina Gillings School of Global Public Health, Chapel Hill, NC, USA; Department of Health Behavior, University of North Carolina Gillings School of Global Public Health, Chapel Hill, NC, USA; Wits RHI, Faculty of Health Sciences, University of the Witwatersrand, Johannesburg, South Africa; Wits RHI, Faculty of Health Sciences, University of the Witwatersrand, Johannesburg, South Africa; Division of Social and Behavioural Sciences, University of Cape Town School of Public Health, Cape Town, South Africa; MRC  MRC  Center for AIDS Prevention Studies (CAPS), Department of Medicine, University of California San Francisco, CA, USA; Wits RHI, Faculty of Health Sciences, University of the Witwatersrand, Johannesburg, South Africa; | US National Institute of Mental Health; Wellcome Trust UK; the South African Medical Research Council; the University of Witwatersrand; Royster Society of Fellows at the University of North Carolina at Chapel Hill; UKaid from the Department of International Development (DfID) through the STRIVE Research Programme Consortium | 2020 | South Africa |
|  | (Bamanyaki and Holvoet, 2016) | Integrating theory-based evaluation and process tracing in the evaluation of civil society gender budget initiatives | Belgium | Belgium | 0 | 2 | University of Antwerp, Belgium University of Antwerp, Belgium | Flemish Interuniversity Council | 2016 | Uganda |
|  | (Cazarin Brito, 2021) | Tactical Activism: Religion, Emotion, and Political Engagement in Gender Transformative Interventions | Spain | NA | 0 | 1 | Universitat Autonoma de Barcelona | Department of Science Policy of the Government of the Basque Country/Eusko Jaurlaritza through the postdoctoral fellowship scheme 2017-2020 | 2021 | South Africa |
|  | (González, 2016) | Refugees and ‘host communities’ facing genderbased violence: developing an area-based approach to gender-based violence around Mbera Camp, Mauritania | Senegal | NA | 0 | 1 | Sahel Region, Senegal. | Not declared | 2016 | Mauritania |
|  | (Skovdal *et al.*, 2022) | How gender norms and ‘good girl’ notions prevent adolescent girls and young women prevent adolescent girls and young women from engaging with PrEP: qualitative insights from Zimbabwe | Denmark | Zimbabwe and UK | 5 | 9 | Department of Public Health, University of Copenhagen, Øster Farimagsgade 5, 1014 Copenhagen, Denmark. Department of Public Health, University of Copenhagen, Øster Farimagsgade 5, 1014 Copenhagen, Denmark. Manicaland Centre for Public Health Research, Biomedical Research and Training Institute, Harare, Zimbabwe. Manicaland Centre for Public Health Research, Biomedical Research and Training Institute, Harare, Zimbabwe. Manicaland Centre for Public Health Research, Biomedical Research and Training Institute, Harare, Zimbabwe. Manicaland Centre for Public Health Research, Biomedical Research and Training Institute, Harare, Zimbabwe. Manicaland Centre for Public Health Research, Biomedical Research and Training Institute, Harare, Zimbabwe. Department of Health Policy, London School of Economics and Political Science, London, UK Manicaland Centre for Public Health Research, Biomedical Research and Training Institute, Harare, Zimbabwe | National Institute of Mental Health under Grant R01MH114562–01; the Bill and Melinda Gates Foundation under Grant ID: INV-009999 (formerly OPP1161471); CN and SG acknowledge joint MRC Centre for Global Infectious Disease Analysis funding from the UK Medical Research Council and Department for International Development under Grant MR/R015600/1. | 2022 | Zimbabwe |
|  | (Triulzi *et al.*, 2022) | Understanding the meanings of male partner support in the adherence to therapy among HIV- positive women: a gender analysis | Italy | Italy | 1 | 9 | Institute of Management, Scuola Superiore Sant’Anna, Pisa, Italy; Gender Center, Graduate Institute of International and Development Studies, Geneva, Switzerland; Dream Programme, Community of Sant’Egidio, Blantyre, Malawi; Institute of Management, Scuola Superiore Sant’Anna, Pisa, Italy; Department of Biomedicine, University of Tor Vergata, Rome, Italy; Dream Programme, Community of Sant’Egidio, Blantyre, Malawi; UniCamillus, Saint Camillus International University of Health Sciences, Rome, Italy; Department of Human Science, Lumsa University, Rome, Italy Institute of Management, Scuola Superiore Sant’Anna, Pisa, Italy; | Italian Ministry of Foreign Affairs through Italian Agency for Cooperation and Development within the ‘Global Fund 5% | 2013 | Malawi |
|  | (van den Berg *et al.*, 2013) | ‘One Man Can’: shifts in fatherhood beliefs and parenting practices following a gendertransformative programme in Eastern Cape, South Africa | South Africa | USA | 5 | 6 | Sonke Gender Justice Network University of the Western Cape University of California at San Francisco, USA Sonke Gender Justice Network Sonke Gender Justice Network Social and Behavioral Sciences at the University of California at San Francisco (UCSF), USA. | National Institutes of Health, University of California, San Francisco, Gladstone Institute of Virology & Immunology Center for AIDS Research, P30-AI027763 | 2015 | South Africa |
|  | (Viitanen and Colvin, 2015) | Lessons learned: program messaging in gender-transformative work with men and boys in South Africa | USA | South Africa | 1 | 2 | Global Health Sciences, University of California San Francisco, San Francisco, CA, USA; School of Public Health and Family Medicine, University of Cape Town, Cape Town, South Africa | Time to work on this article was partially supported by the Eunice Kennedy Shriver National Institute of Child Health & Human Development of the National Institutes of Health (award number R24HD077976). | 2013 | South Africa |
|  | (Adams, Salazar and Lundgren, 2013) | Tell them you are planning for the future: Gender norms and family planning among adolescents in northern Uganda | USA | USA | 0 | 3 | Institute for Reproductive Health, Georgetown University, Washington DC, USA Institute for Reproductive Health, Georgetown University, Washington DC, USA Institute for Reproductive Health, Georgetown University, Washington DC, US | United States Agency for International Development under the terms of the Cooperative Agreement No. AID-OAA-10-00073. | 2020 | Uganda |
|  | (Jewkes and Morrell, 2012) | Sexuality and the limits of agency among South African teenage women: Theorising femininities and their connections to HIV risk practises | South Africa | South Africa | 2 | 2 | SAMRC University of Cape Town | National Institute for Mental Health (grant no.MH 64882-01) | 2012 | South Africa |
|  | (Leight *et al.*, 2021) | Community-level spillover effects of an intervention to prevent intimate partner violence and HIV transmission in rural Ethiopia | USA | USA | 2 | 5 | Poverty, Health and Nutrition Division, International Food Policy Research Institute, Washington, District of Columbia, USA Addis Ababa University School of Public Health, Addis Ababa, Ethiopia CARE, New York, New York, USA EngenderHealth, Addis Ababa, Ethiopia Department of Global Health and Population, Harvard T H Chan School of Public Health, Boston, Massachusetts, USA | anonymous donor, Fondation de France | 2021 | Ethiopia |
|  | (Newmann *et al.*, 2021) | Measuring Men’s Gender Norm Beliefs Related to Contraception: Development of the Masculine Norms and Family Planning Acceptance Scale | USA | USA | 2 | 9 | University of California, San Francisco, CA, USA University of California, San Francisco, CA, USA School of Nursing and Health Studies, University of Washington Bothell, Bothell, WA, USA University of Southern California Institute On Inequalities in Global Health, Los Angeles, CA, USA Kenya Medical Research Institute, Nairobi, Kenya Kenya Medical Research Institute, Nairobi, Kenya University of California, San Francisco, CA, USA Kenya Medical Research Institute, Nairobi, Kenya University of California, San Francisco, CA, USA | Society of Family Planning Research Fund and by the University of California, San Francisco Academic Senate through its Research Allocation Program | 2021 | Kenya |
|  | (Fleming *et al.*, 2016) | What role can gender-transformative programming for men play in increasing men’s HIV testing and engagement in HIV care and treatment in South Africa? | USA | USA | 2 | 4 | Center on Gender equity and Health, Division of Global Public Health, university of California, San Diego, USA Division of Social and Behavioural Sciences, School of Public Health and Family Medicine, university of Cape town, Cape town, South Africa Division of Social and Behavioural Sciences, School of Public Health and Family Medicine, university of Cape town, Cape town, South Africa Department of Social and Behavioral Sciences, School of Nursing, university of California, San Francisco,CA, USA | US national Institutes of Health held at the University of California, San Francisco- gladstone Institute of Virology & Immunology Center for AIDS Research [grant number P30-AI027763]. Paul J. Fleming was supported by the US national Institute of Allergy and Infectious Diseases [grant number T32 AI007001] and subsequently by the US national Institute on Drug Abuse [grant number T32 DA023356]. Shari L. Dworkin was supported by the gladstone Institute of Virology and Immunology, Center for AIDS Research [grant number P-30-AI027763]. Christopher Colvin was supported by the US national Institute of Mental Health [grant number 1R01MH106600-01]. | 2016 | South Africa |
|  | (Nachega *et al.*, 2012) | A Promising Approach to Preventing Gender-Based Violence and HIV Among Slum-Dwelling Youth in Nairobi, Kenya | USA | Kenya | 5 | 7 | University of Nebraska-Lincoln, USA; Life Skills for Behavior Change Center, Nairobi, Kenya; Bennington College, VT, USA Life Skills for Behavior Change Center, Nairobi, Kenya; Life Skills for Behavior Change Center, Nairobi, Kenya; Life Skills for Behavior Change Center, Nairobi, Kenya; Life Skills for Behavior Change Center, Nairobi, Kenya; | The author(s) received no financial support for the research, authorship, and/or publication of this article. | 2021 | Kenya |
|  | (Nyamhanga, Frumence and Simba, 2017) | Prevention of mother-to-child transmission of HIV in Tanzania: assessing gender mainstreaming on paper and in practice | Tanzania | Tanzania | 3 | 3 | Department of Development Studies, Muhimbili University of Health and Allied Sciences, Dar es Salaam, Tanzania; Department of Development Studies, Muhimbili University of Health and Allied Sciences, Dar es Salaam, Tanzania; Department of Community Health, Muhimbili University of Health and Allied Sciences, Dar es Salaam, Tanzania | Research in Gender and Ethics (RinGs): Building Stronger Health Systems, which is funded by the UK Department for International Development (DFID) under UK aid. | 2017 | Tanzania |
|  | (Aventin *et al.*, 2021) | Adaptation of a gender-transformative sexual and reproductive health intervention for adolescent boys in South Africa and Lesotho using intervention mapping | UK | South Africa | 5 | 11 | School of Nursing and Midwifery, Queen’s University Belfast, Belfast, Northern Ireland, UK; Institute for Life Course Health Research, Stellenbosch University, Cape Town, South Africa; Institute for Life Course Health Research, Stellenbosch University, Cape Town, South Africa; School of Nursing and Midwifery, Queen’s University Belfast, Belfast, Northern Ireland, UK; Dreams Project, World Vision, Federal Way, Lesotho Institute for Life Course Health Research, Stellenbosch University, Cape Town, South Africa; School of Nursing and Midwifery, Queen’s University Belfast, Belfast, Northern Ireland, UK; Centre for Public Health, Queen’s University Belfast, Belfast, Northern Ireland Centre for Public Health, Queen’s University Belfast, Belfast, Northern Ireland School of Education, Social Sciences, Education and Social Work, University Belfast, Belfast, Northern Ireland, UK Institute for Life Course Health Research, Stellenbosch University, Cape Town, South Africa | Department for the Economy Northern Ireland under grant number [DfE_ GCRF_19/1920]. | 2021 | South Africa; Lesotho |
|  | (Okedo-Alex, Akamike and Uneke, 2021) | Does Spousal Engagement Improve Cervical Cancer Screening Discussions and Uptake? Lessons from a Before-After Study in a Rural Nigerian Community | Nigeria | Nigeria | 3 | 3 | African Institute for Health Policy and Health Systems, Ebonyi State University, Abakaliki, Nigeria. African Institute for Health Policy and Health Systems, Ebonyi State University, Abakaliki, Nigeria. African Institute for Health Policy and Health Systems, Ebonyi State University, Abakaliki, Nigeria. | Not provided | 2021 | Nigeria |
|  | (Özler *et al.*, 2020) | Girl Empower – A gender transformative mentoring and cash transfer intervention to promote adolescent wellbeing: Impact findings from a cluster-randomized controlled trial in Liberia | USA | USA | 0 | 6 | The World Bank, 1818 H Street, NW, Washington, DC, 20433, United States Population Council, United States International Rescue Committee, United States City University of New York, United State International Rescue Committee, United States International Rescue Committee, United States | NoVo Foundation | 2019 | Liberia |
